# The Neurobiology of Mutualistic Behavior: The Cleanerfish Swims into the Spotlight

**DOI:** 10.3389/fnbeh.2017.00191

**Published:** 2017-10-17

**Authors:** Marta C. Soares

**Affiliations:** CIBIO, Centro de Investigação em Biodiversidade e Recursos Genéticos, Universidade do Porto, Porto, Portugal

**Keywords:** cooperation, cleanerfish, nonapeptides, neurotransmitters, cortisol

## Abstract

One of the most notorious examples of cooperation between different species happens in the cleaner-client fish mutualism. The best known cleaner fish species, the bluestreak Indo-Pacific cleaner wrasse *Labroides dimidiatus* has been a model system to study the evolution of cooperation between unrelated animals and between distinct species during the last couple of decades. Given that the cleanerfish mutualism is well-established for behavioral studies of cooperation, it offered an outstanding opportunity to identify the link between cooperation, social cognition, and to undertake proximate studies, which were severely in need. This review surveys the current achievements of several recent studies, pointing towards the potential of the cleanerfish mutualism as a relevant model system for future accomplishments in neuroendocrine research.

## The Cleaning Mutualisms: Classic Examples of Interspecific Cooperation

Cooperation is a widespread phenomenon that has fascinated biologists for centuries, known to occur either among individuals of the same species or between different species (i.e., mutualism). Typically, cooperation refers to behavioral interactions where the costs (i.e., investment in the behavioral interaction) should be outweighed by the outcome benefits (Dugatkin, 1997; Sachs et al., 2004). But why would any organism choose to invest in another without knowing exactly if it will be (at least) equally beneficial? To answer this, there has been much research on the diversity, evolution and stability of animal cooperative behavior, and a considerable amount of knowledge accrued.

Among social species that are reported to engage in cooperative behavior, those that do so with non-relatives are less frequent. In this respect, human societies excel in the magnitude of cooperative activities and, contrarily to most other animal species, humans frequently cooperate with genetically unrelated strangers, often in large groups, with people they will never meet again (Melis and Semmann, 2010). One rare example of cooperation between unrelated individuals in a non-human model happens in the cleaner-client mutualism between fish. For instance, the best-known example of a cleaner fish species, the Indo-Pacific cleaner wrasse *Labroides dimidiatus* has been a model system to study the evolution of cooperation between different species for the last couple of decades. Cleaner fish inspect the surface, gills and sometimes the mouth of so called “client” reef fish, eating ectoparasites, mucus, scales and dead or infected tissue (Côté, 2000). Individuals of this species, may engage in as many as 2000 such interactions per day (Grutter, 1995). While there is evidence that both cleaners and clients typically gain from their encounters, there is also evidence of several conflicts of interest that lead to the development of sophisticated strategies: for instance, predatory clients may try to eat cleaners, cleaners prefer the protective mucus layer of clients over ectoparasites, and sometimes two or more clients simultaneously seek the service of the same cleaner (Bshary and Côté, 2008). In addition, cleaners are known to recognize individual clients, to distinguish between client categories (predators—non-predators, residents –visitors), to reconcile and manipulate client decisions by providing a form of tactile stimulation with their pelvic fins, to adjust their service quality to the presence or absence of bystander potential clients, and to use predators as social tools to stop other clients from punishing after a bite (Bshary and Côté, 2008).

My major aim in recent years has been to identify neurohormonal candidates that may modulate levels of cooperation in marine cleaning mutualisms but also to test for new experimental paradigms, building on the fact that these are well-established model systems for behavioral studies of cooperation. The cleanerfish system offered a great opportunity to identify the link between cooperation, social cognition, and its mechanistic and neural basis. Studies using cleanerfish as models for cooperative behavior between unrelated individuals are now beginning to provide relevant insights into the neurobiology of cooperation. Here I will review recent discoveries concerning the neuro-endocrine pathways of cleaning behavior and how these interact with the different cognitive dimensions of cleanerfish behavior. Moreover, these studies aim to establish a new model system, useful to understand the proximal mechanisms of other animals, and perhaps even to humans’ cooperative behavior. This animal model may facilitate developing and testing new paradigms on the proximate mechanisms that render altruistic behavior as psychologically rewarding.

## The Cognitive Dimensions of Cleaning Behavior

A few years ago, a manuscript by Soares et al. (2010) proposed the main cognitive modules or building blocks of cooperative behavior as: (i) prosocial behavior; (ii) social recognition; (iii) social bonding; (iv) assessment of social environment; (v) social memory and learning; (vi) temporal discounting; and (vii) partner choice. Building upon this previous description that aimed at general cooperative behavior, I identify the major specific cognitive dimensions present in the cleaning mutualisms (from the cleaner fish perspective, see Table 1), which represent behaviors that enable cleaners to coordinate actions to produce maximum pay-offs, to recognize and evaluate partners and finally, the ability to respond appropriately (Soares et al., 2010). Notably, these dimensions are not mutually exclusive, for instance, they may function in dependence and may also contribute asymmetrically to the regulation of cleaners’ behavior.

**Table 1:** The cognitive dimensions of cleaner fish, their putative underlying social skills and selected species examples in which these were reported to occur.

Cognitive dimension	Social skill	Species	References
**a. Predisposition to approach partners**			
	Advertising dance^ (1)^	Bluestreak cleaner wrasse *Labroides dimidiatus*	Losey (1987)
	Cleaner predisposition to initiate interactions	Caribbean cleaning gobies *elacatinus* spp; Labroides bicolor and *L. dimidiatus*	Soares et al. (2007) and Oates et al. (2010)
**b. Impulsivity and deception**			
	Feeding against preference; temporal discounting^(2)^	*L. dimidiatus*	Grutter and Bshary (2003)
	Interaction termination/partner switching	*L. dimidiatus*	Gingins et al. (2013)
	Interaction termination/Punishment^(3)^	*L. dimidiatus*	Bshary and Grutter (2005)
	Pairs cheating^(4)^	*L. dimidiatus*; *elacatinus* spp	Bshary et al. (2008) and Soares et al. (2009)
	Cheating and home range size	*L. dimidiatus; L. bicolor*	Mills and Côté (2010)
	Third party punishment^(5)^	*L. dimidiatus*	Raihani et al. (2010)
**c. Social recognition and inference**			
	Individual recognition/familiarity	*L. dimidiatus*	Tebbich et al. (2002)
	Partner value assessment	*L. dimidiatus*	Soares et al. (2008)
	Eavesdropping and image scoring^(6)^	*L. dimidiatus*	Bshary (2002) and Bshary and Grutter (2006)
	Audience effects^(7)^	*L. dimidiatus*	Pinto et al. (2011)
**d. Learning and memory**			
	Cue and spatial stimuli learning	*L. dimidiatus*	Cardoso et al. (2015a)
	Reversal learning	*L. dimidiatus*	Salwiczek et al. (2012)
	Pavlovian conditioning^(8)^	*L. dimidiatus*	Soares et al. (2017b)
	Reverse reward contingency^(9)^	*L. dimidiatus*	Danisman et al. (2010)
	Keeping track of when and what	*L. dimidiatus*	Salwiczek and Bshary (2011)
	Repeated interactions delay	*Labroides bicolor*	Oates et al. (2010)
**e. Communication and levels of investment**			
	Single and paired Inspection duration	*L. dimidiatus*	Gingins and Bshary (2014)
	Predatory clients’ inspection duration	*Elacatinus* spp	Soares et al. (2007)
	Tactile stimulation/Predator tactile stimulation provision	*L. dimidiatus*	Bshary and Würth (2001) and Grutter (2004)
**f. Bonding**			
	Pair association	*L. dimidiatus*	Cardoso et al. (2015b)
	Sexual selective dominance	*L. dimidiatus*	Raihani et al. (2012b)
	Partner familiarity	*L. dimidiatus*	Raihani et al. (2012a)

*(1) “Dancing” in cleaner fish serves to advertise cleaning services to potential client fish. (2) Temporal discounting occurs when individuals choose a lower immediate reward to maintain future benefits. (3) Punishment occurs when a client or another cleaner charge and tries to bite, the focal cleaner following a cheat. (4) “Pairs cheating” occurs when 2 cleaners inspect clients together (male-female couples) need to adjust their cheating levels if they want to continue to attract clients. (5) Third party punishment, also known as altruistic punishment, refers to a phenomenon in which a cleaner is punished for cheating a client by an outside observer (another cleaner) who is not directly affected by the violation. (6) Cooperating with another partner increases one’s image score, whereas cheating or refusing to cooperate decreases it. (7) Audience effect or social facilitation is the tendency for cleaner to perform differently when in the presence of others (clients) than when alone. (8) Pavlovian or classic conditioning refers to a learning procedure in which a relevant stimulus or cue (e.g., food) is paired with a previously neutral stimulus (e.g., a bell). (9) In the reversed-reward contingency task, subjects are required to choose the less preferred of two options in order to obtain the more preferred one*.

### Predisposition to Approach Partners

Known as “parasite eaters”, this terminology says very little about cleaners’ behavior. Considering that the foraging patch of any cleaner is another living animal, in practice, this adds a whole dynamic setting to the process. It is also interesting that, for cleaners, the “food” is presented to them; e.g., clients visit cleaners at their cleaning stations. To approach partners successfully, cleaners need to overcome their putative shyness and approach clients, sometimes, even before these clients demonstrate willingness to be cleaned (i.e., when cleaners approach fish that are solely passing nearby). Cleaners’ pro-activity is an incentive for the occurrence of social (between conspecifics, same species) and mutualistic (between cleaners and clients, aka different species) interactions (see Table 1), as these interactions go beyond the simple “foraging behavior”. Indeed, cleaners may engage with clients without gaining any foraging benefits, just to provide tactile stimulation for example, which is variably provided amongst clients (but extremely frequent to predators; Grutter, 2004).

### Impulsivity and Defection

In the cleaning mutualisms, cleaners rely on judgement based on their cognitive toolbox as to choose to cooperate (invest) or to defect (cheat). Another relevant feature of the cleaning mutualisms is the existence of conflict of interests between Indo-pacific cleaner wrasses (*L. dimidiatus*) and clients, but seemingly absent in the Caribbean cleaning gobies (*Elacatinus prochilos*) because these prefer to eat parasites, which is in accordance to clients’ needs (Soares et al., 2010). The existence of conflict is based on cleaners *L. dimidiatus* feeding preference for client-gleaned mucus instead of parasites, which constitutes defection (also known as cheating) as it is detrimental to the clients (Grutter and Bshary, 2003, 2004). Thus, cleaners that prefer to forage on mucus need to learn to eat against their primordial preference if they want to continue to inspect clients and to secure a good reputation (Grutter and Bshary, 2003). However, the temptation to cheat is extremely high because from the gleaning of client mucus, cleaners get additional calories and essential amino acids (Eckes et al., 2015). This predisposition to eat client derived mucus seems to be less evident in Caribbean cleaning gobies and in most of the facultative cleaners, which also defect by eating client derived scales and some mucus, but do not seem to prefer to do so (Soares et al., 2010; Côté and Soares, 2011; Vaughan et al., 2016). The ability to refrain from eating mucus (aka to defect) should ultimately be a mechanism of inhibition of impulsivity, which should be modulated by cleaners’ previous social experiences (learning), and together with their physiological status, gives rise to strategic adjustments of cooperative behavior and tactical deception (Soares et al., 2014; Binning et al., 2017).

### Social Recognition and Inference

For cleaners, social recognition is vital to distinguish between dangerous (predators) and non-dangerous partners and, amongst these, between those that are familiar (frequent visitors with whom they interacted before) and novel partners (Bshary and Côté, 2008). Moreover, cleaners adjust their behavior in accordance to client value (parasite infestation and mucus quality) and choice options (whether clients have access to one or more cleaning stations; Soares et al., 2008). This happens because visitor clients will eavesdrop local cleaners’ behavior (towards other clients) as to gain information and will solely interact with the cleaner if was previously observed to cooperate (Bshary, 2002; Pinto et al., 2011). Hence, this creates a pressure on cleaners’ strategic decision making, with cleaners paying attention to their reputation, particularly when in presence of an audience and if bystanders are of good value (for instance, if clients are large, highly parasitized and covered with high-quality mucus; Bshary and Grutter, 2006; Pinto et al., 2011). Consequently, cleaners use this information to decide how often, whom they should bite under and which circumstances, as to achieve contextual benefits and attract key clientele to be inspected (Bshary and Grutter, 2006).

### Memory and Learning

Cleaner interactions with clients are extremely frequent, with the same individual clients visiting cleaning stations several times each day. For instance, cleaner wrasses *L. dimidiatus* inspect an average 2297 fish clients per day (Grutter, 1995), belonging to many species differing in size, color pattern, parasite infestation and trophic level; which ultimately contributes to characterize clients in terms of value (Côté, 2000; Bshary and Côté, 2008; Vaughan et al., 2016). The ability to learn and to recall previous interactions is decisive for cleaners’ selective adjustments of service. The level of socio-environment complexity seems to underlie the cognitive background of these animals (for instance, their learning abilities, see Wismer et al., 2014), which potentially influence the way clients exert their choice options (Soares et al., 2013). In fact, cleaners’ cognitive abilities have been tested in several different studies (see Table 1) that have demonstrated their remarkable cognitive and cooperative competence, even when directly compared to some primate species (Salwiczek et al., 2012).

### Communication and Levels of Investment

One of the most notable features of cooperation, is the need to invest in one’s partners without knowing the level of outcome benefit. For cleaners, investment in clients comes in various shapes, varying in risk when inspecting a predator (because they may be eaten) and unpredictability, when wanting to inspect a choosy client that may not be willing to wait and leaves as soon as the cleaner forages on mucus instead of parasites (Bshary and Côté, 2008). The amount of time providing service is, for most dedicated and facultative cleaners, the most important commodity being traded; however, for most labroides’ cleaners, the provision of physical contact (referred to as tactile stimulation) is the greatest currency involved (Bshary and Würth, 2001). It has been referenced that these cleaners provide tactile stimulation to build relationships with new clients, to reconcile after a cheating event, to prolong interactions with clients about to leave and as a pre-conflict management strategy with predators (Bshary and Würth, 2001; Grutter, 2004). The provision of physical contact is a costly investment on behalf of cleaners, as they refrain from eating during its provision. Clients on the other hand, benefit from receiving tactile stimulation as it lowers baseline and acute stress levels (i.e., cortisol levels; Soares et al., 2011). Hence, cleaners use the provision of tactile stimulation as a mean of negotiation for the occurrence and duration of cleaning interactions with clients.

### Bonding

Cleaners establish privileged relationships with conspecific partners that contribute to their behavioral variation. Cleaners are sometimes found alone (which happens only for females, in the case of *L. dimidiatus*), but they are usually associated in mixed-sex pairs and sometimes even in larger groups (the case of *Elacatinus* spp; Bshary and Côté, 2008; Côté and Soares, 2011). In the case of the cleaner wrasses *L. dimidiatus*, males are harem holders, live and clean in pairs, usually with the largest female of their harem; however, they will keep on patrolling the other single females regularly (Robertson, 1972). The quality of cleaning service provided to clients can suffer from conflicts that may arise within the couple of cleaners, due to the benefits of cheating, which may solely be secured by one of the cleaners during joint inspections (e.g., the first to cheat will induce the client to leave, Bshary et al., 2008). However, because larger male cleaners may punish (i.e., aggressively chase) the females that cheat, paired inspections are usually of better quality mainly as females behave more cooperatively in joint inspections than during solitary ones (Bshary et al., 2008; Raihani et al., 2010). Thus, a wide variation in cleaning service quality is expected, as a consequence of relationship differences between cleanerfish (couple) associations.

## The Nonapeptide Switch: to Clean or Not to Clean?

**Figure 1:**
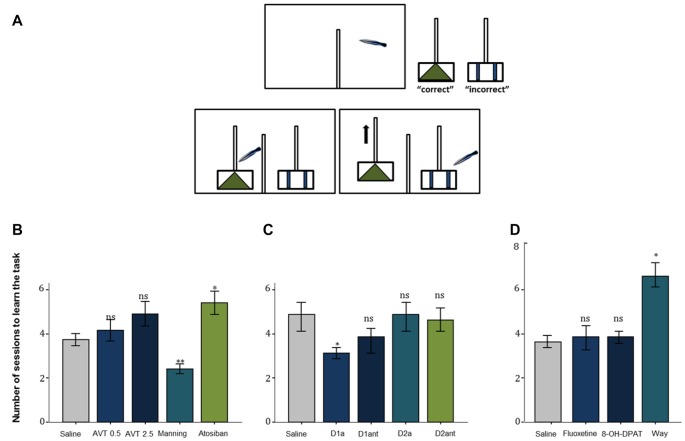
**(A)** The experimental aquarium (view from the front) was divided into a back compartment (right) and the test compartment (left) with help of an opaque PVC partition. The experiments had three stages, an initial phase, a second in which the two plexiglas plates are placed, and if the cleaner chooses the correct pattern/side, both plates will stay in the aquarium, with one piece of shrimp available, and finally the two plexiglas plates are placed, and if the cleaner chooses the incorrect pattern, only the incorrect plate remains, with no shrimp available. The two Plexiglas plates were visually separated from each other with help of a dividing transparent partition wall in-between the plates. The tested fish could move into the test compartment when an opaque partition at the side of the back compartment is pulled up. **(B)** The effect of the neuropeptides arginine vasotocin (AVT), Manning compound (Manning), and atosiban on learning behavior of the cleaner fish *L. dimidiatus* in the ecologically relevant cue-learning task, compared with a control (saline). **(C)** The effect of D1 receptor agonist (D1a), D1 antagonist (D1ant), D2 agonist (D2a) and D2 antagonist (D2ant) on *L. dimidiatus* cue- learning task, compared with a control (saline) and **(D)** The effect of fluoxetine (FLX), 8-OH-DPAT and WAY 100.635 on the number of sessions to learn the cue task, compared with the saline control. Symbols above bars represent *p* values, which refer to planned comparisons of least squares means effect of each compound treatment group against the reference (saline) group (*p < 0.05; **p < 0.01; *ns* > 0.05); error bars represent the standard error of the mean. Cleaners had a maximum of eight sessions to learn the task.

Two neuropeptides emerged initially as strong candidate mediators of cleaning behavior: arginine vasotocin (AVT) and isotocin, which are teleostean homologs of mammalian nonapeptides arginine vasopressin (AVP) and oxytocin (OT) and are critically known for their core physiological and behavioral functions across vertebrate taxa (particularly those related to regulation of social behavior; Acher and Chauvet, 1995; Goodson and Bass, 2001). Intramuscular injection of AVT, made for the first time to cleaners in natural conditions, caused important behavioral changes: cleaners ceased inspecting clients and switched their focus to conspecific activities (Soares et al., 2012a). This significant decrease in the propensity to approach clients in the wild, under the effect of AVT treatment, was then further confirmed in a laboratorial experiment, in which cleaners’ (following AVT infusions) latency to react to confined surgeonfish (inside a smaller aquarium) was comparably higher than to a confined conspecific (Soares et al., 2012a). The mechanism underlying cleaners’ motivational switch (from a higher predisposition to engage in interspecific activities related to foraging, to focussing exclusively on conspecific behaviors) could be due to alterations in central AVT signaling. The hypothesis that changes in AVT activity would lead to a behavioral switch was reinforced when cleaners’ learning skills were tested under the influence of AVT (module d. in Table 1): cleaners were introduced to two different problems (cue and spatial discrimination tasks), one mimicking a situation that occurs regularly with clients (cue task) while the other was less in tune with cleaners’ frequent behavior (spatial task), e.g., cleaners usually choose clients in accordance to pattern (visual cue) and not necessarily to the location of the clients (right or left; see Figure 1A, Cardoso et al., 2015a). While AVT was confirmed to slow cleaners’ ability to learn in both tasks, it was solely in the cue learning discrimination that the V1a antagonist (Manning compound) revealed to have opposite effects (e.g., increased cleaners’ learning speed; Figure 1B; Cardoso et al., 2015a). These results were consistent with previous studies (Soares et al., 2012a; Mendonça et al., 2013), suggesting that lower AVT activity should underlie cleaners’ interest to feed and interact interspecifically, while higher AVT activity may prone cleaners into mating activities (Cardoso et al., 2015a).

Compared to the substantial effects of the AVT motivational switch in regulating the interest in cleaning, the effects on cleaning service quality were less clear (Soares et al., 2012a). Regarding deceptive behavior, it was by blocking the effects of V1a receptors that cleaners seemed to become more impetuous, switching from one client to the next and thus causing clients to jolt more often (Soares et al., 2012a). However, at the level of control of cleaners’ innate impulsive choice, it was solely higher dosages of AVT, that led to a decrease of cleaners’ willingness to feed against preference (Cardoso et al., 2015c). Because the effects of the V1a antagonist were prompting cleaners to interact with more clients, but not necessarily providing better service, it was again demonstrated that higher amounts of AVT would not contribute to a better cooperative judgment. Indeed, female cleaner wrasses with higher levels of forebrain AVT appeared to cheat more (i.e., to more often bite their clients, which jolted in response) during cleaning interactions (Cardoso et al., 2015b). Nevertheless, cleaner wrasse cooperative outcome could be conditioned by other variables that may influence individuals to invest when dealing with partners. For instance, the existence of social ties between cleaner wrasse pairs that inspect clients together, seems to be important, particularly to males (see module f. in Table 1). Cardoso et al. (2015b) found that males living in stronger/stable pair associations received greater amounts of partner support (tactile stimulation and cleaning) from females and exhibited higher levels of forebrain isotocin. Interestingly, these males with higher levels of forebrain isotocin tended to cheat on their clients more often than their female counterparts (Cardoso et al., 2015b).

Considering the strength of AVT as a switch, turning on or off, the very expression of interspecific cooperative behavior in cleaner wrasses, it was further hypothesized that lower levels of AVT could also be a prerequisite for approaching and interacting with clients, being reflected in their AVT neuronal phenotype. Interestingly, comparative neuroanatomical studies revealed minor differences between obligate cleaners *L. dimidiatus* in relation to the closest related species of non-cleaners *Labrichthys unilineatus*: cleaners had smaller and less numerous immuno-reactive AVT neurons in the gigantocellular preoptic area (gPOA) but, no differences between the two species in the number or size of parvocellular POA and magnocellular POA neurons were detected (Mendonça et al., 2013). When measuring the levels of active brain nonapeptide levels, a following comparative study found that, contrarily to what was predicted, obligatory cleaner fish species had overall higher whole brain and cerebellum AVT levels compared to a close related facultative and a non-cleaner species (Kulczykowska et al., 2015). The results generated by these recent efforts aiming at nonapeptidergic crucial influence on cleaners’ decision-making, particularly the identification of the cerebellum as a candidate area underlying the function of AVT on cleaners’ motor and cognitive output and the importance of gPOA cell group, highlighted the need of further integrative studies. For instance, these studies should aim to find out more on how neuropeptides influence cleaners’ central decisions by affecting the dynamic state of the brain Social Behavior Network (SBN).

## The Dopaminergic Mediation in Cleaning

**Figure 2:**
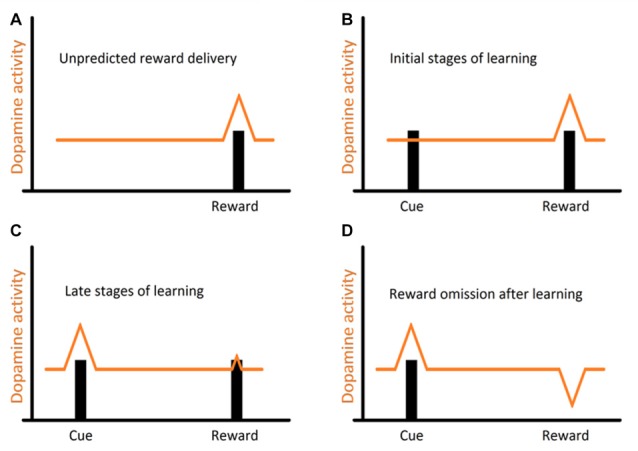
Representation of the evolution of dopaminergic (DA) response (DR) throughout the learning process: **(A)** usual DR to a novel, unpredicted reward; **(B)** a reward is presented after displaying a cue. This allows animals to associate the receipt of a reward to the previously displayed cue; **(C)** after repeated encounters, this DR progressively transfers from the reward itself to the earlier reward-predicting cue, and now animals can expect a reward every time that cue is presented (prediction); **(D)** however when a reward is predicted but fails to occur (reward omission), a reward-prediction error is signaled.

**Figure 3:**
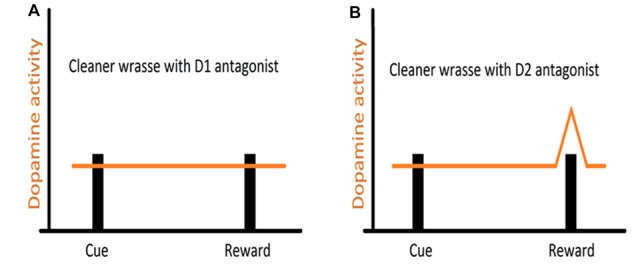
Hypothesized representation of DA manipulation on reward signaling in cleaners: **(A)** DA transmission is disrupted by D1 antagonist administration, causing the DA signaling to fail, even when the reward is achieved, hence the continuous providing of tactile stimulation, even when cleaner wrasses have access to their client’s body surface (hypothetical reward); **(B)** with DA transmission impaired by D2 antagonist administration, the prediction signal that should fire upon cue display is disrupted and fails to occur. However, DA still signals the reward achieved and hence cleaners stop providing tactile stimulation.

Studies, mostly on mammalian models, and more recently across vertebrates, have shown that reciprocity, cooperativeness and social rewards activate reward processing areas with strong dopaminergic (DA) input (for instance, Schultz et al., 1997; Salamone and Correa, 2002; Wickens et al., 2007; Aragona and Wang, 2009; O’Connell and Hofmann, 2011, 2012). However, the involvement of DA extends to animals’ assessment of risk, influencing the perception of each behavioral action (built on the outcome of previous behavioral experiences) either as appetitive or aversive (Schultz, 1998; Salamone and Correa, 2002). Indeed, DA works as a teaching signal, evolving through the learning process, first allowing animals to associate a given cue to a reward delivery and then by progressively enabling animals to anticipate reward, linked to the predicting cue (Schultz, 2002, see Figure 2). Furthermore, when a reward is predicted but fails to occur (reward omission), the reward-prediction error is signaled (Figure 2, Schultz et al., 1997). Thus, DA was put forward as a strong candidate mediator of cleaners’ decision process, predicted to enable the assignment of different value to different clients-type, but mostly to the appraisal of anticipation of reward gain (or risk) associated to each cleaning interaction. The first study, which focused on the inference of exogenous manipulations to the DA system of cleaners, on their predisposition to interact in natural conditions, was solely found effective when decreasing DA transmission aiming at the D1 like receptors (Messias et al., 2016a). Indeed, the effect of DA disruption on cleaners’ proactivity carried an interesting twist: cleaners were approaching and engaging with clients for longer, not necessarily to forage but to provide tactile stimulation (Messias et al., 2016a). It was explained to occur because reductions in DA transmission were signaling an outcome that was worse than predicted, which would anticipate a lower probability of getting food during the course of an interaction or alternatively, a higher likelihood of being punished (by being chased or the client leaving, see Messias et al., 2016a). Interestingly, the actual increase in investment was observed by the blockage of both receptors tested (D1 and D2 like), with D2 receptors causing an increase of tactile stimulation events, but not the amount of time spent providing it; while D1 blockade produced a stronger impairment on cleaner wrasses’ overall behavior (Figure 3). The influence of DA’s D1 receptor-like disruption on cleaners’ provision of tactile stimulation was further tested in laboratorial conditions, revealing to be dependent on the level of familiarity with their partners being highly exacerbated whenever clients are non-familiar, and unnoticed when dealing with familiar ones (Soares et al., 2017b). These overall results of DAergics’ D1-D2 blockage on cleaners’ behavior contrasted with the absence of measurable behavioral effects of DA exogenous increases.

Interestingly, effects of DA pharmacological stimulation were solely evident during the learning process (Schultz, 2006), in which individual learning abilities of cleaners were tested in laboratorial conditions, following a protocol similar to that described in Figure 1A (see Table 1). Evidence indicated a greater role for D1 receptor pathways than D2 receptor pathways in both reward-associative learning tasks, which were putatively dependent on dorsomedial and dorsolateral telencephalon regions (Figure 1C; Messias et al., 2016b). However, when cue and reward are discounted by imposed delays and/or localization (for instance, reward is given some time after, at a different site), then the subjective value of reward may change; which may frequently occur in the context of cooperation, when animals need to choose a lower immediate reward if they want to maintain future benefits (Bshary and Côté, 2008). In this case, DA signaling is thought to be crucial to the levels in which the incentive elicited by a food-related cue is able to shift individual choices (Flagel et al., 2011a,b). To cleaners, a putative temporal and spatial delay between the presentation of a discrete visual stimulus and a food reward was found to predominately favor a “sign tracking strategy” (e.g., cleaners that immediately respond to the conditioned-cue plate, Soares et al., 2017a). Again, the D1 receptor blockage was responsible for reducing cleaners’ sign tracking behavior, nearly to the level of those that were considered as “goal trackers” (cleaners that wait and/or approach the reward plate location instead of responding to the initial cue plate), while the increase of DA levels went on to produce a devaluation of the outcome, by reducing the provision of tactile stimulation to the reward plate (Soares et al., 2017a). Thus, the authors demonstrated that variations of DA signaling (in particular the D1 receptor pathways) modulate cleaners’ incentive properties associated to client-cues, underlying shifts in cleaners’ impulsive behavior and consequent variation in cooperative levels (Soares et al., 2017a). However, in the wild, experimental manipulations in adult cleaners’ DA system, did not affect their levels of cooperative foraging and cheating rates (Messias et al., 2016a). Hence, it is safe to assume that the scope of DA influence should be greater during cleaners’ cognitive development stages (juvenile), for instance, when they learn to prefer some species over others and/or, to eat against their preference as a way to prolong clients’ visiting time (Bshary and Côté, 2008).

## Serotonin: A Powerful Motivational Trigger

Serotonin is another neurohormonal compound that plays a vital role in cooperative behavior, as it is substantially implicated in the basic drive to be social (Fox et al., 2009; Young, 2013). Studies regarding serotoninergic influence to complex social behavior have mostly focussed on humans and other mammalian models, covering a broad set of themes, from prosociality to anti-social impulsive related disorders and aggressive behavior (for example: Wood et al., 2006; Crockett et al., 2008; Fox et al., 2009; Coccaro et al., 2010; Bilderbeck et al., 2011). However, most studies using fish models to test for serotonin influence have focused on the role of serotonin in social status and aggression (Clotfelter et al., 2007; McDonald et al., 2011), with few exceptions (Beulig and Fowler, 2008; Paula et al., 2015; Soares et al., 2016). Profiting from cleaners’ unique complex system, the first tests were again done in natural conditions, which enabled the examination of the neuromodulatory role of serotonin in interspecific cleaning behavior, as well as in general social behavior between conspecifics. Predictably, the clearest results were found on cleaners’ predisposition to approach clients (module a., see Table 1), with the administration of a selective serotonin reuptake inhibitor fluoxetine and a serotonin 1A receptor agonist leading to an overall increase in the motivation interact with clients, whereas the blocking of serotonin-mediated effects had the opposite effect (see Paula et al., 2015). The effect of the decrease of serotonin levels took a significant toll on all remaining cleaning service measures (related to cleaners’ levels of investment and deception) causing a lowering of cheating frequencies due to an overall reduction of the proportion of clients inspected and of the average duration of interactions (Paula et al., 2015). Moreover, in laboratorial conditions, the reduction of serotoninergic signaling, via administration of receptor 1A antagonist went on to produce a slowing of learning speed in comparison with saline treated fish (Figure 1D; Soares et al., 2016). As discussed by Soares et al. (2016), the effects of serotonin depletion may occur via mediation of risk perception, which would enhance their anxiety, fear appraisal and, perhaps even, their aggressiveness in relation to clients. An increase of aggressive behavior was solely observed towards conspecifics, which were mostly lower status females. No doubt that these effects maybe occurring in association to other neuroendocrine systems (affecting the Hypothalamic-Pituitary-Interrenal axis and/or involving the AVT system), but at this stage is still difficult to know. Furthermore, the overall effect of the serotonin 1A receptor agonist 8-OH-DPAT on tactile stimulation duration provides strong links between the connection of these results with the dopaminergic system (Messias et al., 2016a,b) but again via a potential rise in the interrenal stress response, i.e., an increase in cortisol production (Winberg et al., 1997; Höglund et al., 2002). The latter will be further discussed below.

## The Role of Stress-Related Mechanisms

The first few studies aiming at the physiological effects of interacting with cleaner organisms, found indications for stress reduction and immune benefits arising from client-cleaner interactions (Bshary et al., 2007; Ros et al., 2011). From the cleaner perspective, a first behavioral approach suggested a role for putative short term (acute) stress on cleaners’ service quality: in the wild, clients would be found to jolt less following a cleaner-predator interaction, while in laboratorial conditions, cleaners altered their foraging behavior by increasing cooperative levels (eating more against preference, which in the wild would mean more parasite removal) when in presence of a stressor (an hand-net, see Bshary et al., 2011). However, the hypothesis that stress mechanisms are involved remained to be confirmed as no physiological measures were collected. Work with Caribbean cleaning gobies *Elacatinus evelynae* provided the first insights on how stress response mechanisms may underlie the high prosociality of cleaners. Cleaning gobies became more proactive towards predatory than towards herbivorous clients, and reduced the time elapsed between client approach and the start of client inspection—this was associated with interrupting the potentially harmful physiological consequences (cortisol levels rise) caused by the approach of predatory clients (Soares et al., 2012a). In addition, these cleaners spent more time inspecting dangerous clients, even though these clients offered no obvious foraging advantage (i.e., ectoparasites; Soares et al., 2007), perhaps to enforce a reduction of cortisol levels by securing a good outcome to the cleaning event (Soares et al., 2012b).

The role of stress-related mechanisms on the modulation of cleaners’ levels of cooperation (aka investment and cheating) was then further tested in natural conditions, focusing on cleaners’ ability to switch between behavioral tactics (see Bshary, 2002). Cleaners responded to an exogenous increase of cortisol levels by providing more tactile stimulation to smaller resident clients, which attracted larger bystanders that were then bitten (see above module c. in Table 1); while the effect of the glucocorticoid antagonist led to the opposite effect with more investment to larger clients (Soares et al., 2014). This was a clear demonstration of the crucial influence of cortisol-associated mechanisms on cleaner decision-making process, between cooperating and cheating; and applying a different behavioral tactic (e.g., tactical deception). However, because tactical deception is a sophisticated social strategy, very much dependent on interspecific social complexity, the following question focused on how far would these learned decision rules based on social habitat differences (whether cleaners are interacting with a higher or lower client diversity) alter the scope for physiological modulation. By subjecting cleaners from high vs. low social complexity sites to cortisol treatment, Binning et al. (2017) found that only those from high complexity habitats used tactical deception as a function of the cortisol treatment. Thus, the scope of endocrine modulation seems to be dependent on cleaners’ socio-cognitive landscape, as a mechanism underlying pre-acquired context-dependent strategic behavioral adjustment (Binning et al., 2017).

## Cleaning Behavior: The Need for a Symphony of Neuro-Modulators?

**Figure 4:**
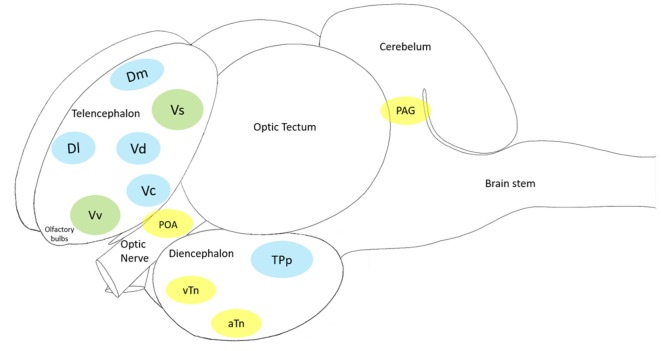
The cleaner fish *Labroides dimidiatus* brain schematics, showing the main macroa-areas: olfactory bulbs, telencephalon, diencephalon, optic tectum, cerebellum and brain stem. In blue: the Mesolimbic Reward System (MRS); in yellow: the Social Behavior Network (SBN) and in green: the Social Decision-Making Network (SDN—MRS + SBN). Each of the 11 brain nuclei (showed in color) has a putative mammalian counterpart: the medial zone of the dorsal telencephalic area (Dm, putative homolog of the mammalian basolateral amygdala) and the lateral zone of the dorsal telencephalic area (Dl, putative homolog of the mammalian hippocampus), the preoptic area (POA), the ventral nucleus of the ventral telencephalic area (Vv, putative homolog of the mammalian lateral septum) and the supracommissural nucleus of the ventral telencephalic area (Vs, putative homolog of the mammalian medial extended amygdala and the bed nucleus of the stria terminalis), the dorsal part of the ventral telencephalon (Vd, putative homolog of the mammalian nucleus accumbens), the central part of the ventral telecephalon (Vc, putative homolog of the mammalian striatum), the periventricular nucleus of the posterior tuberculum (TPp, putative homolog of the mammalian ventral tegmental area), the ventral tuberal nucleus (vTn, putative homolog of the mammalian anterior hypothalamus), the anterior tuberal nucleus (aTn, putative homolog of the mamalian ventromedial hypothalamus) and the midbrain periaqueductal gray (PAG, putative homolog of the mammalian periaqueductal gray).

**Figure 5:**
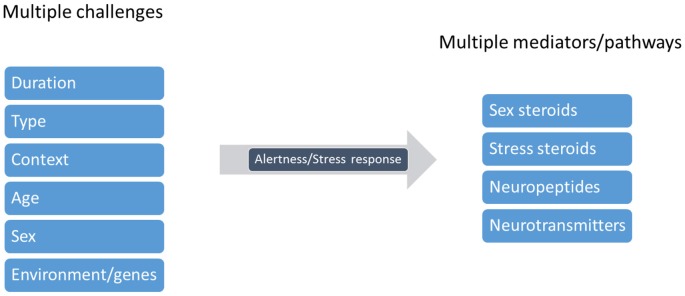
Cleaning behavior warrants a neuro-symphony of modulators. Cleaners are subject to several challenges that will modulate the state of alertness or rather, cleaners’ stress response. These factors include the duration (pontual vs. chronic), type (heterospecific or conspecific), context (for instance, client threatens to leave), age (the developmental stage of the cleaner), cleaner sex and its genetic or environmental background. Various substances may thus be released in response to each challenge, and may influence distinct neural pathways, acting alone or combined, affecting cleaners’ decision making (figure adapted from Joëls and Baram, 2009).

**Figure 6:**
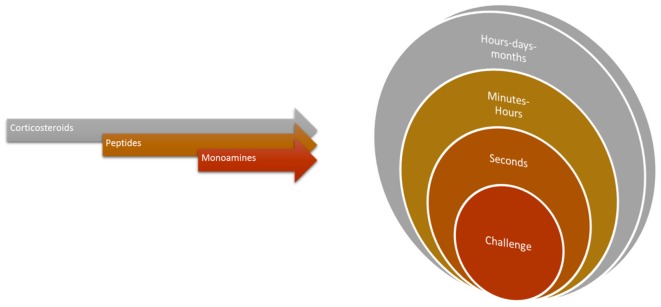
The temporal profiles of the orchestrated physiological response demanded of cleaning behavior. For instance, receptor activation by monoamines, peptides or corticosteroids can have rapid stimulation (or blockage) within seconds to minutes (synaptic or membrane located effects) to genomic or more structural effects happening in wider temporal intervals.

Cleaners’ social landscape is notoriously diverse and dynamic (Bshary and Côté, 2008; Côté and Soares, 2011; Vaughan et al., 2016). Potentially, habitats’ social complexity, (for instance, client fish diversity and cleaner-cleaner competition) required of cleaners’ central machinery (brain function) the ability to deal with an entire range of putative stress-related challenges, which in principle gave way to cleaners’ sophisticated behavioral output (see Figure 4). Challenges as those described above, such as receiving a visit from a predatory client or having an important bystander nearby, require immediate attention from cleaners and question their strategical decisions, as to gain access to new food resources. These challenges evoke the need to storage key information that allows for cleaners to recognize and react appropriately in future repeated encounters. Stress mechanisms may be triggered by a myriad of social challenges, raising the need for different mediators that will act on different brain areas and time scales (Joëls and Baram, 2009). The challenge of cooperation, and of cleaning behavior, warrants for the active use of multiple instruments, multiple mediators that will orchestrate cleaners’ brains remarkable ability to respond and adapt to a dynamic environment. The effect of a multi-array of factors, as summarized in Figure 5, together with others, such as season, circadian rhythm, creates a complex matrix of pathways, which could be under the effect of cortisol and of stress response. For example, animals’ life stage and experience may influence the valence of any signal being perceived as a stressor, as the mediators that are released and their consequences (Fenoglio et al., 2006; Shors, 2006). This orchestration would depend on the deployment of a high repertoire of signaling molecules that can bring about temporal, spatial and context specificities to each individual behavioral response (see Figure 5, Joëls and Baram, 2009). For instance, rapid actions would come from monoamines, such as serotonin, dopamine, nor-adrenaline (Figure 6, Orchinik et al., 2009). These rapid actions would directly promote vigilance, alertness, appraisal to any given situation, memorization and choice of the optimal strategy in face of a challenge. Peptides, such as AVT, may also be of short temporal action (as monoamines) but have also a medium sustained effect (Landgraf and Neumann, 2004), thus modulating more adaptive components of stress response and behavior; components that may alter an individual predisposition to cooperate or not. Finally, long lasting effects would mostly be accomplished by actions of corticosteroids, perhaps through alterations of gene expression and cell function; however, these corticosteroids may also rapidly modulate brain functioning (Joëls and Baram, 2009).

Although our understanding of cleaner fishes’ proximate mechanisms has increased substantially, usually by testing isolated effects per neuro-endocrine system, we still have little knowledge on the combination of effects and how for instance, stress may change the scope of each modulator’s influence on short and long-term behavior. More studies are needed, focusing on the coordinative action of neuro-modulators, in several spatial and temporal frames. Future work will certainly bring us exciting new avenues of research concerning this and other fascinating cooperative model systems.

## Author Contributions

MCS is the sole contributor.

## Conflict of Interest Statement

The author declares that the research was conducted in the absence of any commercial or financial relationships that could be construed as a potential conflict of interest.
